# Multi-omic analysis of meningeal cerebral amyloid angiopathy reveals enrichment of unsubstituted glucosamine and extracellular proteins

**DOI:** 10.1093/jnen/nlaf018

**Published:** 2025-03-29

**Authors:** Joshua E Mayfield, Alexander J Rajic, Patricia Aguilar-Calvo, Katrin Soldau, Samantha Flores, Roger Lawrence, Biwsa Choudhury, Majid Ghassemian, Donald P Pizzo, Steven L Wagner, Garrett A Danque, Paige Sumowski, Lawrence A Hansen, Vanessa Goodwill, Jeffery D Esko, Christina J Sigurdson

**Affiliations:** Department of Pathology, University of California, San Diego, La Jolla, CA, United States; Department of Pathology, University of California, San Diego, La Jolla, CA, United States; Neurology Service, Veterans Affairs Medical Center, La Jolla, CA, United States; Department of Pathology, University of California, San Diego, La Jolla, CA, United States; Department of Pathology, University of California, San Diego, La Jolla, CA, United States; Department of Pathology, University of California, San Diego, La Jolla, CA, United States; Department of Cellular and Molecular Medicine, University of California, San Diego, La Jolla, CA, United States; Department of Cellular and Molecular Medicine, University of California, San Diego, La Jolla, CA, United States; Department of Chemistry and Biochemistry, University of California, San Diego, La Jolla, CA, United States; Department of Pathology, University of California, San Diego, La Jolla, CA, United States; Department of Neurosciences, University of California, San Diego, La Jolla, CA, United States; Department of Pathology, University of California, San Diego, La Jolla, CA, United States; Department of Pathology, University of California, San Diego, La Jolla, CA, United States; Department of Pathology, University of California, San Diego, La Jolla, CA, United States; Department of Pathology, University of California, San Diego, La Jolla, CA, United States; Department of Cellular and Molecular Medicine, University of California, San Diego, La Jolla, CA, United States; Department of Pathology, University of California, San Diego, La Jolla, CA, United States; Department of Medicine, University of California, San Diego, La Jolla, CA, United States; Department of Pathology, Microbiology, and Immunology, University of California, Davis, Davis, CA, United States

**Keywords:** Alzheimer's disease, glycomics, heparan sulfate, neurodegeneration, protein misfolding, proteomics

## Abstract

Cerebral amyloid angiopathy (CAA) is a common feature of Alzheimer’s disease in which amyloid-β (Aβ) deposits in cerebral and leptomeningeal vessel walls, predisposing vessels to micro- and macro-hemorrhages. The vessel walls contain distinct proteins and heparan sulfate (HS), yet how vascular proteins and HS jointly associate with Aβ is unknown. We conducted the first multi-omics study to systematically characterize the proteins as well as the HS abundance, sulfation level, and disaccharide composition of leptomeninges from 23 moderate to severe CAA cases and controls. We then analyzed the associations between Aβ and other proteins, HS, and apolipoprotein E genotype. We found an increase in a minor HS disaccharide containing unsubstituted glucosamine, as well as 6-O sulfated disaccharides; Aβ40 levels positively correlated with unsubstituted glucosamine. There was also an increase in extracellular proteins derived from brain parenchyma or plasma, including olfactomedin-like protein 3, fibrinogen, serum amyloid protein, apolipoprotein E, and secreted frizzled related protein-3. Our findings of vascular HS and protein alterations specific to CAA-affected leptomeningeal vessels provide molecular insight into the extracellular remodeling that co-occurs with Aβ deposits and may indicate a basis for antemortem diagnostic assay development and therapeutic strategies to impede Aβ-HS interactions.

## INTRODUCTION

Alzheimer’s disease (AD) is the most common cause of age-related neurodegeneration, affecting over 30% of adults aged 85 and older.^1^ Cerebral amyloid angiopathy (CAA) is common in AD patients (80%-90%), with a reported incidence of 30% in the sixth decade and 50% by the seventh decade.^2^ In CAA, Aβ initially deposits within the basement membrane and then the tunica media of cerebral and leptomeningeal vessels. Further, Aβ deposition causes fibrinoid necrosis, hyaline degeneration, and microaneurysmal dilatation,^3^ compromising vascular integrity and increasing the risk of micro- or lobar hemorrhage.^4^ Additionally, the recent use of anti-Aβ immunotherapies has commonly led to vascular inflammation, increased vascular permeability and edema, and microhemorrhages visible as amyloid-related imaging abnormalities (ARIA), particularly in patients with CAA. This response to immunotherapy can present clinically similar to patients having an inflammatory form of CAA.^5–7^

Early analysis of Aβ parenchymal plaques demonstrated a strong colocalization of proteoglycans, particularly heparan sulfate proteoglycans (HSPGs).^8–11^ HS is a linear anionic glycosaminoglycan composed of alternating subunits of d-glucuronic (GlcA) or l-iduronic acid (IdoA), and *N*-acetyl-d-glucosamine (GlcNAc), which are modified after polymerization by a series of GlcNAc N-deacetylases-N-sulfotransferases and O-sulfotransferases. The chains assemble while covalently attached to a proteoglycan core protein, which can be transmembrane, GPI-anchored, or secreted into the extracellular matrix.^12^^,^^13^ Most proteins that bind to HSPGs, such as growth factors, cytokines, and morphogens, bind to small sections of the HS chains dictated by the pattern of sulfation.^14^ HSPGs are enriched in the basement membrane adjacent to vascular endothelial cells,^11^^,^^15^ at the site of early Aβ binding in vessels.

Although CAA is common, the role of HS in initiating CAA is incompletely understood. HS binds many protein ligands in the extracellular space or on membranes, in part through electrostatic interactions with the negatively charged sulfate groups.^14^ HS binds Aβ residues 12-18^16^^,^^17^ and accelerates Aβ fibril formation,^18^ as well as mediates Aβ internalization and toxicity in vitro.^19–21^ In mouse models of AD, neuronal HS depletion of the HS polymerization enzyme, exostosin 1 (Ext1), reduced parenchymal Aβ plaques and accelerated Aβ clearance, suggesting that HS enhances Aβ plaque formation in the brain.^22^ In patients with AD, HS was increased by more than 9-fold in the hippocampus,^11^ and HSPGs have been found to be increased in vascular basement membranes.^23^^,^^24^ An LC-MS/MS analysis of AD-affected brain homogenate revealed that the disaccharide composition of HS in AD brain samples (frontal cortex) had markedly higher levels of a specific 3-*O*-sulfated HS vs controls.^25^

While HS levels have been studied in CAA, the relative abundance of disaccharide moieties and sulfation is unknown, and HS and protein composition have not been previously compared in the same CAA vessel samples. Several previous AD studies characterized differences in the HS enzyme gene expression with conflicting results,^26^^,^^27^ suggesting that RNA transcript levels may not clarify the pathogenic events driving CAA.^28^^,^^29^ Here, we use a mass spectrometry approach to directly determine the HS composition and proteome in the same leptomeninges from patients having AD-related CAA and non-CAA controls. We found HS and protein components were altered in CAA-affected leptomeninges, including an increase in vascular clotting proteins and proteins of emerging interest in CAA such as olfactomedin-like protein 3 (OLFML3) and secreted frizzled related protein-3 (FRZB). We discovered associations between OLFML3 and the disaccharide, D0H0. Notably, the HS disaccharide composition differed, as CAA cases showed significantly higher levels of unsubstituted glucosamine residues, suggesting that specific HS patterning is altered in CAA-affected vessels.

## METHODS

### Cases

Human brains from patients with CAA and control participants were provided by the UCSD ADRC Neuropathology Core following review and approval by the ADRC Biospecimen Review Committee. All human samples were obtained from participants who consented to autopsy by the UCSD ADRC. All tissues provided were de-identified and provided with the demographic data and apolipoprotein E (ApoE) genotype. The cohort consisted of 12 CAA cases with sporadic AD and 11 control cases. The neuropathologic diagnoses, including CAA, Braak staging, CERAD, and Thal phase, were based on gross and histologic examination of multiple brain regions and were determined by neuropathologists (L.A.H. and V.G.) from the ADRC.

### Histology and immunolabeling

Five-micrometer sections of formalin-fixed paraffin embedded brain samples were cut onto positively charged silanized glass slides and stained with hematoxylin and eosin (H&E), or immunostained using antibodies against Aβ (Clone NAB228; ThermoFisher, Waltham, MA, USA; 1:10 000). Immunohistochemical labeling was performed on an automated tissue immunostainer (Ventana Discovery Ultra, Ventana Medical Systems, Santa Clara, CA, USA). Antigen retrieval was performed by incubating the slides in CC1 (tris-based; pH 8.5; Ventana) at 95°C for 40 min. Following retrieval, antibodies were incubated on the tissue for 32 min at 37°C. The secondary antibody (HRP-coupled goat anti-mouse; OmniMap system; Ventana) was incubated on the sections for 12 min at 37°C. The primary antibody was visualized using DAB as a chromogen followed by hematoxylin as a counterstain. Slides were rinsed, dehydrated through alcohol and xylene, and coverslipped.

### Dual immunofluorescent labeling of cortical sections for Aβ and heparan sulfate

For the dual immunofluorescence experiment, epitopes were exposed by sequential treatment with formic acid and then heating in a citrate buffer (pH 6). The sections were blocked and incubated with anti-Aβ 6E10 antibody (BioLegend; 1:300) for 1 h followed by IgG-Cy3 (Jackson ImmunoResearch; 1:500) for 30 min. Sections were next incubated with anti-HS 10E4 antibody (AMS Bioscience, Cambridge, MA, USA; 1:300), anti-mouse IgM biotin (Jackson ImmunoResearch; 1:500) for 30  min, streptavidin-HRP (Invitrogen, Waltham, MA, USA; 1:100) for 45  min, and tyramide-Alexa488 (Invitrogen) for 10 min. Nuclei were labeled with DAPI, and slides were mounted. Single sections immunostained for Aβ or HS and healthy cases were also included as isotype immunoglobulin controls.

For the dual immunofluorescence experiment in which slides were stained for anti-Aβ and anti-HS having unsubstituted glucosamine units (JM-403),^30^ epitope retrieval was performed as described above. Slides were quenched in 3% hydrogen peroxide in PBS, blocked in 5% goat serum with 0.1% Tween20 in PBS, and incubated with anti-HS (JM-403) (1:600, mouse IgM, AMSBIO, 370730) and anti-Aβ (rabbit, Cell Signaling Technology, Danvers, MA, USA: D12B2, 1:1000) overnight, then with CY3 anti-rabbit (1:500). Slides were then incubated in biotin-anti-mouse IgM (1:500) (115-065-075) for 30 min, followed by streptavidin-HRP (1:500) (Jackson ImmunoResearch, West Grove, PA, USA; 016-030-084) for 30 min and tyramide-Alexa-488 (Invitrogen, B40953; 1:100) in a tyramide amplification buffer (Biotium) for 10 minutes. Slides were coverslipped using Prolong Gold antifade medium (Invitrogen).

Images were acquired using the Eclipse Ti2-E (Nikon, Tokyo, Japan) AXR confocal microscope. Aβ and heparan sulfate immunolabeled blood vessels were acquired using the laser scanning confocal mode (A1R HD, Nikon) and Plan Apo ʎ 60× NA 1.40 oil, WD 130 µm and Plan Apo ʎ 100× NA 1.45 oil objectives, respectively, to collect 3.0 μm Z-stacks (Piezo Z-drive, Z step size = 0.2 μm, 15 steps). All imaging functions were integrated into the NIS elements software (version 5.42.02: High Content Analysis package). The 3.0 μm Z-stack images were deconvolved using analysis functions integrated into the NIS Elements software.

### Meningeal homogenization and protein extraction

Meninges were gently removed from cerebral cortices (frontal, parietal, temporal, and occipital) of 23 individuals with CAA or control participants. CAA cases were chosen based on CAA severity (all moderate to severe), while minimizing concurrent non-AD brain disease. Control brain samples were chosen to match age and sex. The meninges from each patient were frozen in liquid nitrogen and then minced. Tris-HCl (100 mM, pH 8.0) was added and the tissue was homogenized using a polytron homogenizer.

### Aβ ELISA

The meningeal homogenates were solubilized under increasing denaturing conditions. Homogenates in PBS were incubated at 37°C with 0.25% sarcosyl (final) for 30 minutes, and then centrifuged at 18 000 × g at 4°C for 30 minutes. The pellet was resuspended in 10% SDS in PBS, incubated for 30 minutes at room temperature, sonicated, and centrifuged at 18 000 × g for 30 minutes. The supernatant was then collected (SDS-soluble Aβ), and the pellet was washed in PBS, and resuspended in 70% formic acid, incubated for 15 minutes at room temperature, sonicated, and neutralized with 2 M Tris to a final pH of approximately 7.5, and centrifuged at 18 000 × g for 30 minutes. The supernatant was collected (formic acid-soluble Aβ). The Aβ40 and 42 levels in the SDS- and formic acid-soluble fractions were then measured by ELISA in a 96-well plate by using either an Aβ40-specific coating monoclonal antibody (B113) or an Aβ42 select monoclonal antibody (A387) coupled with an anti Aβ1-12 alkaline phosphatase conjugated antibody (B436)^31^ (Aβ antibodies kindly gifted from Neurogenetics) followed by CSPD-Sapphire luminescence substrate to detect bound Aβ alloforms using a GloRunner Microplate luminometer (Turner BioSystems, Sunnyvale, CA, USA). Aβ1-40 or Aβ1-42 peptide was used to generate a standard curve.

### Sample preparation for mass spectrometry of peptides

Meningeal tissue homogenates were lysed in an equal volume of 0.5% N-lauryl sarcosine in PBS (pH 7.5) (0.25% final) and incubated with agitation at 37°C for 30 minutes. Samples were then centrifuged at 18 000 × g at 4°C for 30 minutes. The supernatant was discarded, and the pellet washed 6 times in PBS and centrifuged at 18 000 × g at 4°C for 30 minutes. The pellet was resuspended in 10 M urea in 100 mM Tris-HCl (pH 8.0), incubated with agitation at 37°C for 30 minutes, and diluted with 100 mM Tris-HCl to a final concentration of 4 M urea. Samples were then treated with dithiothreitol (1.2 μg/μl final) for 20 minutes, iodoacetamide (8 mM) for 15 minutes, prior to addition of CaCl_2_ (1.6 mM) and digestion with trypsin (Trypsin gold; Promega, Madison, WI, USA) for 48 h at 37°C. The peptides were extracted and desalted using C18 desalting columns (Thermo Scientific, PI-87782).

### Mass spectrometry analysis of peptides

Trypsin-digested peptides were analyzed by ultra-high pressure liquid chromatography (UPLC) coupled with tandem mass spectroscopy (LC-MS/MS) using nano-spray ionization.^32^^,^^33^ The nano-spray ionization experiments were performed using a TripleTOF 5600 hybrid mass spectrometer (ABSCIEX) interfaced with nanoscale reversed-phase UPLC (Waters corporation nano ACQUITY) using a 20 cm- to 75-µm ID glass capillary packed with 2.5-µm C18 (130) CSH beads (Waters corporation). Peptides were eluted from the C18 column into the mass spectrometer using a linear gradient (5%-80%) of ACN (Acetonitrile) at a flow rate of 250 μl/minute for 1 h. The buffers used to create the ACN gradient were as follows: Buffer A (98% H_2_O, 2% ACN, 0.1% formic acid, and 0.005% TFA) and Buffer B (100% ACN, 0.1% formic acid, and 0.005% TFA). MS/MS data were acquired in a data-dependent manner in which the MS1 data were acquired for 250 ms at m/z of 400 to 1250 Da and the MS/MS data were acquired from m/z of 50 to 2000 Da. The independent data acquisition parameters were as follows: MS1-TOF acquisition time of 250 ms, followed by 50 MS2 events of 48 ms acquisition time for each event. The threshold to trigger MS2 event was set to 150 counts when the ion had the charge state +2, +3, and +4. The ion exclusion time was set to 4 s. Finally, the collected data were analyzed using Peaks Studio 12 (bioinformatics solutions) for peptide identifications.

### Proteomics data analysis

Proteomics data analysis and visualization was performed in R. Unmodified label free quantitation values were initially reviewed and samples were excluded if they had quantitation for less than 100 Uniprot IDs or amyloid precursor protein (APP, UniProt accession: P05067) was not detected. 6 CAA cases and 4 control samples were considered in the final analysis. Data were normalized using a Variance Stabilization Normalization approach as implemented by the R-package *vsn2* with an ordinary least sum of squares regression (lts.quantile = 1). Missing values were imputed utilizing a left-censored deterministic minimal value approach as implemented in the R-packages *MsCoreUtils* and *imputeLCMD*. The minimal value observed was estimated as being the qth quantile (q = 0.01) of the observed values in that sample. Data related to non-human proteins, including BSA and trypsin, were not considered during imputation. Original and imputed values were utilized in subsequent statistical analysis. Imputed values are indicated where applicable. Proteins in the original dataset were identified by UniProt accession numbers and annotated with gene symbols, protein names, and UniProt review status using the R-package *Uniprot.ws*. Data relating to unreviewed UniProt accessions were not utilized in the final analysis. Statistical analysis was performed using non-parametric Wilcoxon rank-sum testing of normalized and imputed values between CAA and control cases as implemented by the R-package *rstatix*. Heatmap clustering and visualization was performed using the R-package *stats-package*. Hierarchical clustering was performed using a complete-linkage method as implemented by the R-package *stats-package*. Statistical significance was defined as a Wilcoxon rank-sum test *P*-value less than .05. Ontology analyses were performed on statistically significant proteins using the R-package *clusterProfiler* using the human protein encoded in the R-package *org*. *Hs.eg.db* (version 3.19.1) as background. Gene/protein identifiers were converted as necessary for *clusterProfiler* using the R-package *org*. *Hs.eg.db*. Correlation of protein abundance and HS disaccharide or demographic data was performed using Pearson’s correlation as implemented by the R-package *rstatix*. Data were visualized using base R and R-package *ggplot2*.

### Heparan sulfate preparation for LC-MS analysis

Meningeal homogenates were treated with nucleases (benzonase and MgCl_2_) followed by 0.5 M NaOH (final) and then incubated for 3 h to degrade proteins. The samples were then neutralized with 0.5 M acetic acid (final) and digested with pronase for 25 h at 37°C, to liberate glycosaminoglycans, and then filtered through an 0.20 µm filter. Heparan sulfate (HS) was then extracted by anion exchange chromatography with diethyl-aminoethyl (DEAE) sepharose (Healthcare Life Sciences, Marlborough, MA, USA) as previously described.^34^

### Heparan sulfate analysis by LC-MS

Purified HS (0.9-2 µg) was digested with 1 milli-unit each of heparin lyases I, II, and III to depolymerize the HS chains into disaccharides. HS disaccharides were then tagged by reductive amination with [^12^C_6_]aniline^12^ and mixed with known amounts of [^13^C_6_]aniline-tagged disaccharide standards. Samples were analyzed by liquid chromatography-mass spectrometry (LC-MS) using an LTQ Orbitrap Discovery electrospray ionization mass spectrometer (ThermoFisher Scientific). Internal disaccharides were identified based on their unique mass and quantified relative to their corresponding differentially labeled standards.^34^^,^^35^

### Statistical analysis

Means of 2 groups were compared using an unpaired, 2-tailed *t*-test with Welch’s correction when group sizes varied. Data (summary) are shown as mean ± SEM. Statistical analysis was performed using Prism 10 (GraphPad Software) or R. For all analyses, *P* ≤ .05 was considered significant. Statistical analyses of age, sex, and APOE genotype in Table 1 were performed between control and CAA groups using Welch’s t-test, Fisher exact test, or Mann-Whitney test, respectively. Correlation analyses were performed using linear regressions for continuous independent variables and ordinal regressions for non-continuous independent variables. ApoE score was calculated by assigning a value of −2 for an ɛ2 allele, 0 for an ɛ3 allele, and +1 for an ɛ4 allele. A multivariate linear regression was performed to test the impact of ApoE ɛ4 and Aβ on the HS disaccharide composition.

**Table 1.** Demographics of normal controls and subjects with severe CAA.

Study group	**Age** ^a^	**Sex** ^b^	**APOE genotype** ^c^	**CAA score** ^d^	Braak stage	CERAD	**Thal phase** ^e^	**NIAA-AA ADNC** ^f^	Concurrent disease
**Control**									
N1	93	F	ɛ3ɛ3	0	0	C0	0	Not AD	None
N2	83	F	ɛ2ɛ4	0	0	C0	0	Not AD	Mild focal atherosclerosis^g^
N3	77	F	ɛ3ɛ3	0	0	C0	2	Low	None
N4	92	M	ɛ3ɛ4	0	I	C0	0	Not AD	Moderate multifocal atherosclerosis; chronic microinfarct
N5	89	F	ɛ2ɛ3	0	II	C0	0	Not AD	Trace focal atherosclerosis
N6	81	M	ɛ3ɛ3	0	I	C0	0	Not AD	None
N7	90	M	ɛ2ɛ4	0	II	C1	2	Low	Moderately severe atherosclerosis
N8	90	F	ɛ3ɛ3	0	II	C2	3	Low	Subdural hematoma, 2° brainstem hemorrhages; mild atherosclerosis
N9	86	M	ɛ3ɛ4	0	I	C0	0	Not AD	Mild focal atherosclerosis
N10	62	F	ɛ2ɛ4	0	I	N/A	N/A	N/A	Mild atherosclerosis
N11	80	F	ɛ3ɛ3	0	I	C0	0	Not AD	Mild to moderate multifocal atherosclerosis
*Summary*	84 ± 9.0	7F/4M							
**CAA**									
CAA1	81	F	ɛ4ɛ4	3	V	C3	≥4	High	Arteriolar sclerosis
CAA2	77	M	ɛ4ɛ4	3	V	C2	≥4	High	Subacute and chronic microinfarcts
CAA3	76	M	ɛ4ɛ4	3	VI	C3	≥4	High	None
CAA4	89	F	ɛ4ɛ4	3	V	C2	≥4	High	Multiple neocortical infarcts; mild to moderate atherosclerosis
CAA5	91	M	ɛ4ɛ4	3	V	C2	≥4	High	None
CAA6	84	F	ɛ4ɛ4	3	V	C3	≥4	High	Moderately severe atherosclerosis
CAA7	94	M	ɛ2ɛ3	3	I	C2	3	Low	Moderately severe atherosclerosis
CAA8	97	F	ɛ3ɛ4	3	IV	C3	5	Intermediate	Chronic microinfarcts
CAA9	90	M	ɛ3ɛ4	3	IV	C2	≥4	Intermediate	Chronic microinfarct; hippocampal sclerosis; atherosclerosis
CAA10	96	F	ɛ3ɛ4	3	V	C3	≥4	High	Moderately severe atherosclerosis
CAA11	97	F	ɛ3ɛ4	3	IV	C1	3	Intermediate	Moderately severe atherosclerosis
CAA12	70	F	ɛ3ɛ4	3	VI	C2	≥4	High	None
*Summary*	87 ± 9.1	7F/5M							

Summary statistics listed at the bottom of each column include mean age ± standard deviation, and female to male ratio. Diagnosis of AD was determined according to NIA-AA criteria based on Braak stage, CERAD score, and Thal phase. N/A, not available.

a *P* = .44, Welch’s t-test.

b *P* > .999, Fisher exact test.

c *P* = .0031, Mann-Whitney test.

d 0 = none; 3 = severe.

e Thal phase assigned based on Aβ immunolabeling of neocortex, entorhinal cortex, basal ganglia, and midbrain. For those ≥4, cerebellar amyloid staining was not available.

f ADNC: Alzheimer’s disease neuropathological change.

g Atherosclerosis determined by gross examination of the circle of Willis.

For proteomic analysis, nonparametric tests were applied to compare 2 groups using a Wilcoxon rank-sum test. Correlation tests involving proteomic abundances were performed using Pearson’s method. For all analyses involving proteomics, *P* ≤ .05 was considered significant.

## RESULTS

### Characteristics of the patient population

The study cohort (n = 23) consisted of 11 controls (7F, 4M) and 12 cases of moderate to severe CAA (6F, 6M), ranging in age from 62 to 97 years (Controls: 84 ± 9; CAA: 87 ± 9 years) (M ± SD) (Table 1). Atherosclerosis was common in both groups. Of the CAA cases, 92% (11 of 12) carried an ApoE ɛ4 allele (ɛ4/ɛ4: 6; ɛ3/ɛ4: 5) vs 40% of the control population. Most CAA cases also had abundant parenchymal plaques, a high Braak stage (IV—VI), CERAD score, and Thal phase (10 of 12 cases were 4 or higher), indicating that most cases had advanced AD (Table 1). One CAA case had a low CERAD (C1) and moderate Thal phase^3^ (CAA11), while another CAA case had low AD neuropathologic change (ADNC) by NIA-AA criteria^36^ (CAA7) due to a low Braak score (I). All remaining CAA cases met criteria for intermediate ADNC (3 cases) or high ADNC (8 cases). Control cases were characterized as not AD by NIA-AA criteria (7 cases) or low ADNC (3 cases) [full ADNC staging was not available for 1 control case (N10)].

### Vascular pathology

**Figure 1.**
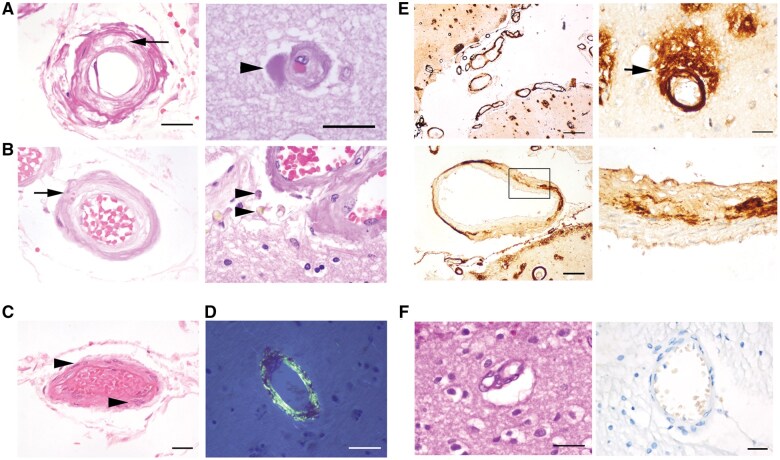
Severe cases of CAA in human brain (cerebral cortex). (A) H&E of vascular lesions showing vacuolation in the tunica media of an arteriole (left image, arrow) (parietal cortex). (Right panel) Amyloid deposits are within and extend beyond the wall of a capillary (dark pink, arrowhead) (temporal cortex). (B) Erythrocytes are visible in the wall of a vacuolated arteriole (small arrow, left), and hemosiderin-laden macrophages indicate a previous microbleed (arrowheads, right) (temporal cortex). (C) Vessel from normal control for reference. Note the higher cellularity in the vessel wall (purple nuclei, arrowheads) compared to the vessel in panel B (occipital cortex). (D) Aβ stained with Congo red (green) (temporal cortex). (E) Aβ immunolabeling of vessels from CAA cases show that Aβ infiltrates and extends outward from arteries and arterioles (arrow). Lower panels show higher magnifications of a vessel in the upper left panel. Early Aβ deposits (dark brown) are visible in the tunica media (temporal cortex). (F) Control samples lack amyloid (H&E) and Aβ in vessel walls (right) (frontal cortex). Scale bars: A, C, D, E (in E, the right upper and lower panel are the same magnification) = 50 µm; in B, the right and left panels are the same magnification as in A right and left, respectively; E, upper and lower left panels, respectively, = 500 and 200 µm.

The vascular pathology in CAA cases was characterized by varying degrees of thickened, hyalinized blood vessel walls with intracellular vacuolation, loss of smooth muscle cells, fibrinoid necrosis, and double barrel lumens. Intracortical vessels occasionally showed Aβ spreading from vasculature into the adjacent neuropil (dyshoric changes) (Figure 1). Leptomeningeal (hereafter designated “meningeal”) vessels did not show overt hemorrhage; however, occasional vessels had erythrocytes within the vessel wall (Figure 1). Meningeal vessels were infiltrated with amyloid, which varied from deposits localized segmentally within the tunica media to global expansion and replacement of the vessel wall (Figure 1).

### Aβ40 and Aβ42 levels

**Figure 2.**
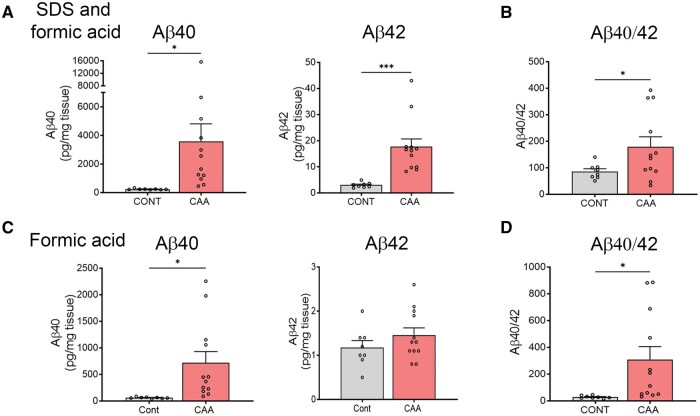
Elevated Aβ40 and Aβ42 levels in meninges from CAA patient samples. (A) Aβ40 and Aβ42 and (B) Aβ40/Aβ42 measured from SDS and formic acid fractions, and (C) Aβ40 and Aβ42 and (D) Aβ40/Aβ42 measured from formic acid only fractions as measured by ELISA. Bar graphs represent the mean ± SEM. Unpaired, 2-tailed *t*-test with Welch’s correction. *P < .05 and ***P < .001.

Meninges were gently removed from the cortex and homogenized. An SDS-soluble fraction was extracted, followed by solubilization of the SDS-insoluble fraction in formic acid (FA). We then measured Aβ40 and 42 levels in both the SDS- and FA-soluble fractions by ELISA from a sample subset (8 controls and 12 CAA) (Figure 2A). Notably, there was a 15-fold increase in total Aβ40 in the CAA samples (SDS and FA fractions) (Con: 242 ± 15 vs CAA: 3576 ± 1230 pg/µg; *P *<* *.05), and a 6-fold increase in Aβ42 as compared to controls (Con: 3.0 ± 0.34 vs CAA: 17.7 ± 3.0 pg/µg; *P* < .0005). Interestingly, for the FA soluble fraction, there was a 12-fold increase in Aβ40 (average) (Con: 62 ± 5 vs CAA: 717 ± 213 pg/µg; *P* < .01), yet no difference in Aβ42. The Aβ40:42 ratio was markedly higher in the CAA cases (11-fold) (FA only: Con: 29 ± 4 vs CAA: 310 ± 97; *P* < .05; SDS + FA: Con: 86 ± 10 vs CAA: 180 ± 37; *P* < .05) (Figure 2B), indicating Aβ40 as the major component of the vascular fibrils. This is consistent with published CAA studies.^37^

### Proteomic analysis

**Figure 3.**
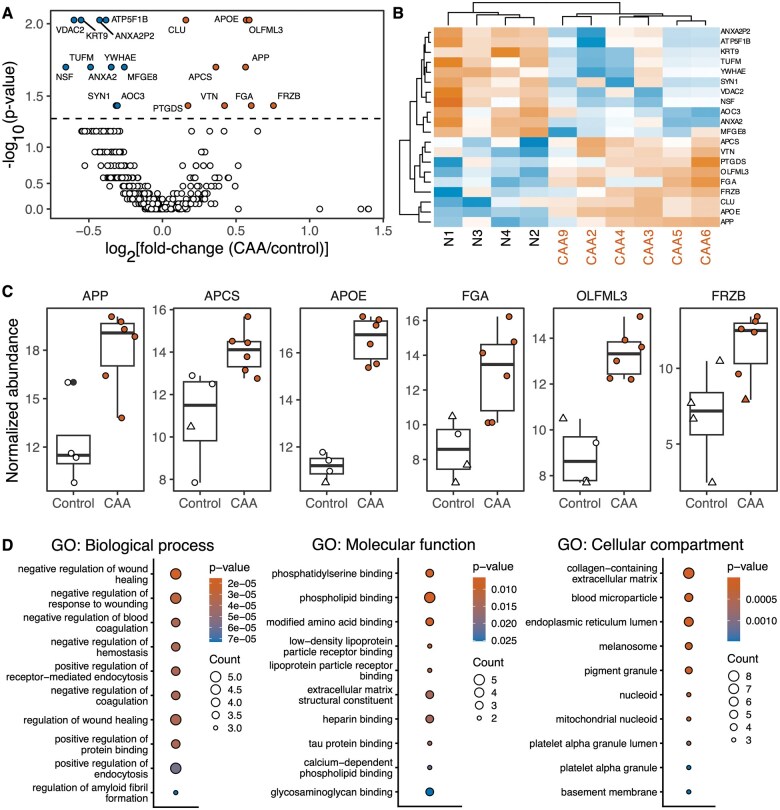
Proteomic analysis of control and CAA meninges. (A) Volcano plot of log_2_ fold changes of protein abundance in CAA vs control cases and the −log_10_ of Wilcoxon rank sum test *P*-value. The y-axis is exponentially transformed (base = 2) to facilitate visualization of individual points. Horizontal dashed line indicates −log_10_(α), where α  =  0.05. Protein abundances increased in CAA relative to control indicated in vermillion. Protein abundances decreased in CAA relative to control indicated in blue. Statistically different proteins labeled. (CAA n = 6, Control n = 4; values and analysis provided in Table S1, tab “Abundance”). (B) Hierarchically clustered heat map of samples against significantly different protein abundances (gradient: blue = lower in CAA relative to control, vermillion = higher in CAA relative to control, white = unchanged). Protein gene symbol indicated along right-hand side of heatmap. Sample identifier indicated along bottom of heat map. Control cases labeled in black font. CAA cases labeled in vermillion font. (C) Box plot of selected differentially abundant genes in control and CAA cases. Values resulting from imputation shown as triangles, non-imputed values shown as circles. All proteins have a Wilcoxon rank-sum test *P*-value less than .05. Box plots of all significantly different proteins are provided in Figure S1. (CAA n = 6, Control n = 4; values and analysis are provided in Table S1, tab “Abundance”). (D) GO ontology analysis of differentially abundant proteins. Adjusted *P*-value indicated by point color. Gene count indicated by point size. All significant ontologies, gene lists, and statistical values are provided in Table S1, tabs 2-4.

To further characterize meningeal alterations at the protein level, we performed LC-MS/MS and label-free quantification of meningeal proteins and analyzed a subset of 10 cases chosen based on proteome coverage and the detection of Aβ peptides (n = 4 control, n = 6 CAA). We identified 862 unique proteins across selected cases, with 20 significantly altered between CAA and control cases (Wilcoxon rank-sum test *P*-values between groups of less than .05) (Figure 3A, Figure S1, Table S1, tab “Abundance”). There were 9 proteins increased and 11 decreased when comparing CAA to control protein abundances and CAA and control cases clustered along these differentially abundant proteins (Figure 3B).

Notably, there were 5 differentially abundant proteins previously linked to CAA or AD either directly or by associated pathway involvement^38–44^: amyloid precursor protein (APP), serum amyloid P-component (APCS, also known as SAP), ApoE (APOE), fibrinogen alpha (FGA), olfactomedin-like protein 3 (OLFML3), and secreted frizzled related protein-3 (FRZB) (Figure 3C).

The APP peptides detected were all derived from the Aβ40 or Aβ42 peptide region of APP (amino acid residue numbers 672 to 711 (Aβ40) or 713 (Aβ42), and at least one peptide was detected in each CAA or control case (Figure S2). As expected, overall APP peptides were increased in CAA cases relative to controls (Figure 3C). Increased SAP abundance was also observed in CAA cases, consistent with SAP being a component of amyloid deposits.^42^ We also observed an increase in ApoE, which co-aggregates with Aβ and modulates Aβ toxicity.^45–47^ There was also increased blood coagulation factor FGA in CAA cases, potentially due to microhemorrhages of fragile, amyloid-laden vessels (Figure 3C).^48^ Collectively APP, SAP, ApoE, and FGA capture key pathological features of CAA at the molecular level and validate these proteomic data.

We also detected an increase in two relatively understudied proteins: olfactomedin-like protein 3 (OLFML3) and secreted frizzled related protein-3 (FRZB). OLFML3 has been reported in multiple AD and CAA proteomic datasets,^38^^,^^43^^,^^44^ and FRZB is linked to the Wnt pathway and has not been previously observed as altered in CAA, but is increased in the serum of AD patients.^41^^,^^49^

Ontological analysis of differentially abundant proteins revealed that top ranked biological process ontologies (by *P*-value) included wound healing and blood coagulation, consistent with CAA-associated vascular lesions. The top molecular function ontologies included lipid-, heparin-, and glycosaminoglycan-binding. The cellular compartment ontologies included extracellular matrix and basement membrane as well as blood microparticles and platelets (Figure 3D). The KEGG pathway analysis revealed signaling pathways implicated in complement and coagulation, consistent with microhemorrhage (Figure S3).

### Heparan sulfate and Aβ localization in vessels

**Figure 4.**
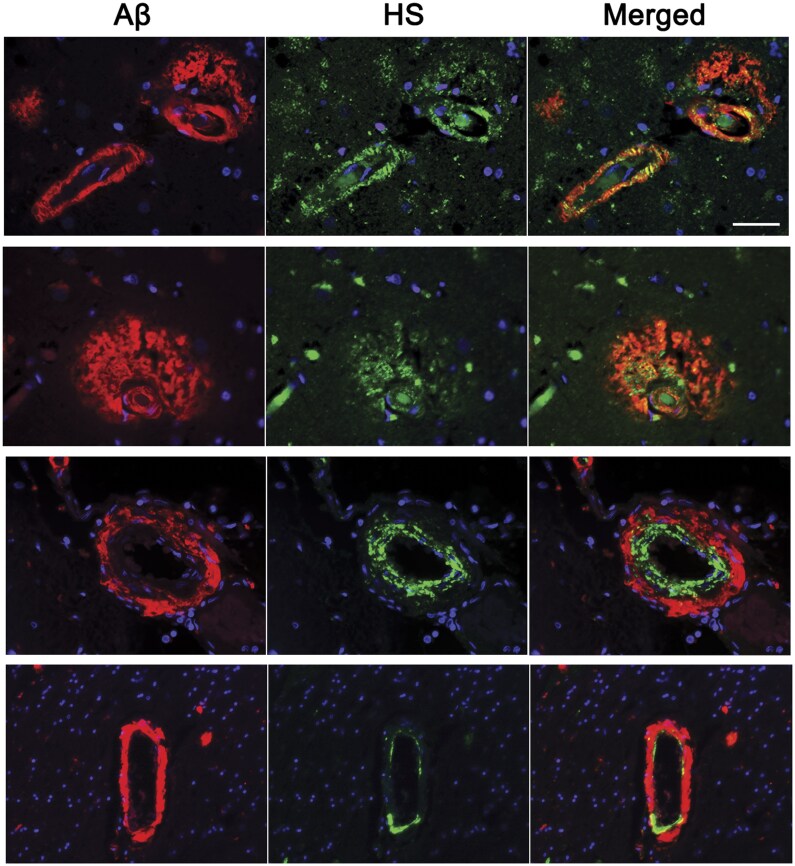
Aβ and HS co-immunolabel CAA-affected vessels. Representative images from 2 CAA cases (top 3 rows: CAA2; bottom row: CAA5) showing HS (anti-HS antibody 10E4) localized adjacent to the tunica intima and media, and Aβ (anti-APP antibody 6E10) localized subjacent to the HS in the tunica media and adventitia. Scale bar: 50 µm.

To test whether HS and Aβ co-localized within the vasculature, we performed immunofluorescent labeling of HS and Aβ in cortical brain sections. Aβ and abundant HS were adjacent and sometimes colocalized within the same vessel (Figure 4). HS immunolabeling was predominantly adjacent to the endothelium and smooth muscle in the arterioles, consistent with basement membrane. Notably, Aβ deposited abluminally, often extending from the HS toward the vascular adventitia (Figure 4).

### Heparan sulfate analysis reveals increased unsulfated glucosamine in CAA cases

**Figure 5.**
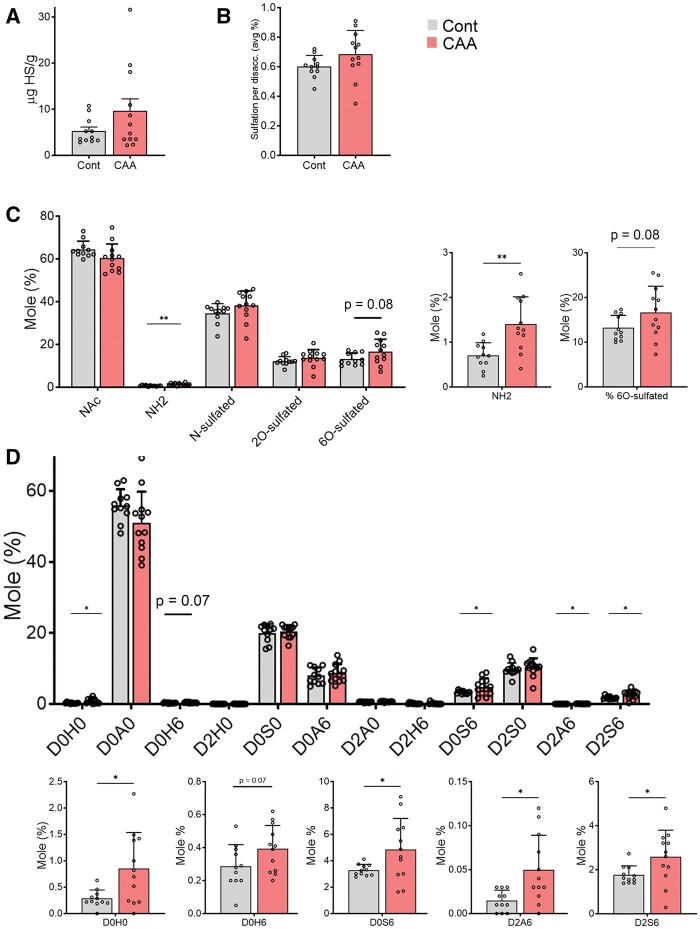
Meningeal HS compositional analysis of CAA cases and controls by LC-MS/MS. (A) Total HS (*P* = .14) and (B) sulfation level per disaccharide (*P* = .12) were quantified. (C) The HS group was quantified and revealed differences in the unsubstituted glucosamine (NH2) and 6-O sulfated HS (see right two panels). (D) Individual disaccharides were quantified, revealing differences in D0H0, as well as the 6-O sulfated disaccharides, D0H6, D0S6, D2A6, and D2S6 (see lower graphs). Bar graphs represent the mean ± SEM. Unpaired, 2-tailed *t*-test with Welch’s correction. *P < .05 and **P < .01.

To quantify the levels and composition of HS, meningeal samples from all cases were homogenized using a polytron homogenizer, and proteins were degraded with proteases and sodium hydroxide. HS was purified and disaccharides cleaved using heparin lyases prior to mass spectrometry analysis.^50^ The average total HS level was increased in the CAA cases but differences were not statistically significant; there was high variability among the samples (Figure 5A, HS Con: 5.3 ± 0.8; CAA: 9.6 ± 2.6 µg HS/g). The average HS sulfation per disaccharide was higher in the CAA samples but differences compared to controls were not significant (Figure 5B, Con: 0.60 ± 0.02; CAA: 0.69 ± 0.05 sulfate per disaccharide, *P *=* *.13).

We next assessed the HS disaccharide composition to broadly compare the mole percentages of N-, 2-O, and 6-O sulfated disaccharides (abbreviation and disaccharide structure key provided in Table S2). Interestingly, the most striking and significant difference was in the unsubstituted glucosamine containing disaccharides (GlcNH_2_), which were 2-fold higher (by mole percentage) in the CAA group relative to controls (Figure 5C). GlcNH_2_-bearing disaccharides are thought to arise during biosynthesis by the action of an N-deacetylase-N-sulfotransferase, when the GlcNAc residue has been N-deacetylated, but not N-sulfated.^51^ Additionally, 6-O-sulfated HS trended higher in the CAA group (*P* = .08).

Comparing the percentages of the individual disaccharides revealed a 3-fold increase in the D0H0 disaccharide, underlying the elevated unsubstituted glucosamine (GlcNH_2_) in the CAA group (Figure 5D). Notably, there was also a striking 5-fold increase in D2A6, which is N-acetylated, 2-O-sulfated, and 6-O-sulfated. The additional elevated disaccharides (by mole percentage) included D0S6, D2S6, as well as a trending increase in D0H6 (*P* = .08). Collectively, the increases in D2A6, D0S6, and D2S6 are suggestive of elevated HS 6-O-sulfotransferase activity (HS6ST) in CAA meninges or reduced expression of endo-6-sulfatases (*SULF1* and *SULF2*). Interestingly, HS6ST is one of the few HS biosynthetic enzymes that are secreted extracellularly, partly regulated by β-secretase.^52^

### Localization of unsubstituted glucosamine HS subunits and Aβ deposits

**Figure 6.**
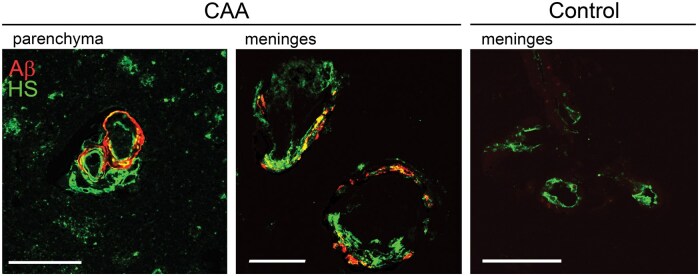
Aβ and HS (JM403) dual labeled vessels. Representative sections of cerebral cortex from a CAA case (CAA10; inferior parietal cortex) and control case (N1; midfrontal cortex) immunolabeled for Aβ and unsubstituted glucosamine-rich HS (JM403). The JM403-immunolabeled HS in vessels was adjacent to the tunica intima, whereas the Aβ deposits extended from the HS into the tunica media and adventitia. The unsubstituted glucosamine-rich HS appeared more abundant in CAA vessels as compared to the non-CAA control sections. Scale bar: 50 µm.

To next localize and characterize HS with unsubstituted glucosamine, we dual immunolabeled sections of cortex for Aβ and JM-403, an antibody previously shown to bind N-unsubstituted glucosamine residues.^53^ JM-403 stained the vessel in a pattern that resembles the endothelial basement membrane and appeared abundant in the CAA cases (Figure 6). Although there was co-labeling of Aβ and HS, most Aβ deposited adjacent and abluminal to the unsubstituted HS (Figure 6).

### Correlation analysis of differentially abundant proteins

We identified changes in both the proteome and HS disaccharides of CAA patient meninges. To test whether the differentially abundant proteins correlated with significantly altered HS disaccharides or demographic variables (age, atherosclerosis, Aβ42 and Aβ40 levels, and Aβ40/42 ratio), we performed a correlation analysis (Figure S4, HS and demographic values provided in Table S1, tab “HS+Demo”). We found a significant (*P*<* *.05) positive correlation between proteomic APP levels and Aβ40 and Aβ42 peptides by ELISA (APP vs Aβ40: r = 0.67; APP vs Aβ42: r = 0.72), as expected. Interestingly, ApoE and OLFML3 also significantly (*P *<* *.05) positively correlated with Aβ peptides (ApoE vs Aβ42: r = 0.72; OLFML3 vs Aβ40: r = 0.67; and OLFML3 vs Aβ42: r = 0.75), suggestive of a co-accumulation or increased abundance in response to Aβ aggregates. Also notable, we found a significant (*P* < .05) positive correlation between APP, ApoE, FGA, FRZB, and OLFML3 and unsubstituted glucosamine (APP: r = 0.72; ApoE: r = 0.67; FGA: r = 0.74; FRZB: r = 0.71; and OLFML3: r = 0.84). Within unsubstituted glucosamine, ApoE correlated with D0H6 disaccharide (r = 0.75), while OLFML3 correlated with D0H0 (r = 0.66), which may indicate an interaction between specific HS disaccharides and proteins within the CAA meninges. Importantly, none of the significantly altered proteins correlated with atherosclerosis, suggesting that the meningeal changes were specific to CAA (Table S3).

### Aβ40 and ApoE genotype correlation with HS composition

To determine the relationship between meningeal Aβ40 and HS disaccharide levels, linear regressions were performed (Figure S5). We found strong direct correlations between Aβ40 and the most highly increased disaccharide, D0H0 (*P* < .01, *r*^2^ = 0.40), but not with any 6-O-sulfated disaccharide, which were unaltered. Interestingly, ApoE ɛ4 allele carriers had a 2.4-fold increase in D0H0 abundance compared to non-carriers (*P* = .02) (Figure S5). Ordinal regression against a computed ApoE risk score showed a strong relationship between D0H0 abundance and the presence of either ɛ2 or ɛ4 ApoE alleles (*P* < .01, *r*^2^ = 0.28) (Figure S5).

Given the correlation of Aβ40 levels and ApoE ɛ4 status with HS composition, we next tested the contributions of these 2 variables on HS composition when considered together. We performed multi-variate linear regression for all significantly altered disaccharides (Table S4). This analysis showed that Aβ40 level was the best predictor of unsubstituted glucosamine level (*r*^2^ = 0.67, *P* = .0015), while ɛ4 dose was the best predictor of 6-O sulfated disaccharides, specifically for D2A6 (*r*^2^ = 0.577, *P* = .0027; ɛ4 dose *P* = .001; Aβ40 *P* = .53). This suggests that HS composition differences may be independently driven by Aβ40 and ApoE ɛ4 levels.

## DISCUSSION

In CAA, Aβ assembles and expands on vascular basement membranes, compressing and replacing smooth muscle, disrupting vascular integrity and raising the risk of micro- and lobar hemorrhages.^3^^,^^54^ Vascular basement membranes contain HSPGs that bind Aβ40 and enhance aggregation in vitro.^17^^,^^55^^,^^56^ The vascular basement membrane is a unique microenvironment in which proteins and proteoglycans are altered in disease states, including AD and CAA.^23^ Here, we used an LC-MS/MS based glycomics and proteomics approach to define how HS and protein composition are modified in CAA-affected meninges. We then analyzed the association of meningeal HS sulfation and protein composition with patient age, co-morbidities, apolipoprotein ɛ4 expression, and Aβ40 and 42 load.

The most striking difference between the CAA and control meningeal HS was in the individual disaccharide subunits. In CAA, there was a highly significant doubling in a minor HS component, unsubstituted glucosamine (0.7% vs 1.4% by mole percentage), which is normally present in the vasculature.^30^ This increase was largely driven by D0H0. Notably, Aβ40 levels directly correlated with D0H0 levels but not with total HS or other disaccharides. In further support of a role for this minor HS subtype, we show that Aβ and HS containing unsubstituted glucosamine residues localize to CAA-affected vessels. Although a minor component, the D0H0 may influence protein binding and aggregate assembly within the vascular basement membrane as it imparts a positively charged unsubstituted amino group within the chain.

Sulfation was reported to be a critical determinant for HS binding to aggregated Aβ^57^ and differentially structured HS co-localizes with Aβ in AD.^58^ Although the HS sulfation levels trended higher in the CAA meninges, there was no significant difference between the groups. A high sulfation dependence may be a unique feature of parenchymal Aβ plaques, but not CAA amyloid, and may explain differences in parenchymal and vascular aggregate composition.^37^^,^^43^

Sulfated disaccharides are typically clustered within the carbohydrate chain, with the sulfate pattern being crucial for HS chains to bind ligands.^14^^,^^59^^,^^60^ Given the significant differences in disaccharide subunits observed for CAA, our data suggest that the specific disaccharide composition is more relevant to CAA than total sulfation. Interestingly, we observed significantly higher 6-*O* sulfated disaccharides, D0S6, D2A6, and D2S6, in the CAA meninges, suggestive of more activity of the sulfotransferase, HS6ST, or reduced activity of the Sulfs. This finding is consistent with prior in vitro work reporting that 6-*O*-sulfated moieties are important for HS to bind Aβ fibrils.^17^^,^^61^ Additionally, prior studies using immunostaining show Aβ preferentially binds HS disaccharides sensitive to the action of SULF1 and SULF2, such as those containing 6-O-sulfated groups.^62^ Sulfate moieties, particularly O-sulfation, have been reported to impact Aβ binding and enhance fibril formation in vitro.^18^^,^^21^ An important future direction is to understand how sulfate micropatterning of HS influences Aβ deposition and aggregate assembly in both CAA- and AD-affected brains. Also of interest is the elucidation of how the increase in D0H0 and 6-O sulfated disaccharides impact Aβ accumulation and vascular inflammation.

Our proteomic analysis of meninges revealed 20 differentially abundant proteins in CAA vs controls, many of which overlap with prior proteomic reports. Recent proteomic studies of AD employed laser capture microdissection to compare proteins in parenchymal Aβ plaques with unaffected parenchyma from early onset AD and Down syndrome cases^38^ or from CAA-affected and unaffected vessels.^44^ A comparison of the proteins that accumulate in our meningeal CAA dataset with those reported for parenchymal Aβ plaques^38^ reveals 6 common proteins: APP, ApoE, SAP, clusterin (CLU), OLFML3, and prostaglandin D2 synthetase (PTGDS), 5 of which also overlap with proteins reported for microdissected CAA-affected vessels.^44^ Vitronectin (VTN) was common to the present study and to the microdissected CAA vessels.^44^ Our analysis of meninges revealed two uniquely increased proteins: fibrinogen A alpha chain (FGA) and FRZB, suggestive of alterations specific to CAA-affected meninges. A limitation of the current study is the bulk analysis of meninges, which precludes the ability to distinguish between vascular and non-vascular contributions to the proteomic differences observed.

Our proteomic analysis also revealed an increase in OLFML3 in CAA-affected meninges. Given that prior proteomics studies also report enrichment of OLFML3 in Aβ plaques relative to surrounding parenchymal tissue in early-onset AD and Down syndrome,^38^ and in CAA-affected vessels captured by laser microdissection,^43^ the current work adds further support to this protein as potentially important in CAA and AD. OLFML3 is a secreted scaffold protein that has been suggested to influence neuroinflammation and angiogenesis.^63–65^ Although molecular mechanisms involving OLFML3 in CAA are not characterized, its consistent appearance in CAA vessels and Aβ plaques, together with emerging recognition of importance in neuroinflammation, warrant further investigation as a molecular marker of CAA and potentially as an extracellular mediator of pathology.

Increased secreted frizzled-related protein 3 (FRZB) was detected the CAA-affected meninges and has not been previously reported in CAA. Recent data indicate that FRZB is increased in heparin-enriched plasma proteins from AD patients.^66^ FRZB is a Wnt-binding protein whose homologs bind Wnt-8 and compete with the Wnt-receptor, frizzled^49^; recent evidence suggests that Wnt signaling decreases in aging and neurodegenerative disease.^39^^,^^41^ Interestingly, FRZB is also implicated in vascular processes such as protection against aortic aneurysms.^67^^,^^68^ Precisely how this potentially protective effect impacts CAA vascular alterations is unknown but the ability of FRZB to modulate Wnt signaling, as well as influence vascular processes, places this protein at an interesting nexus that may be specific to CAA.

We found strong associations between CAA and HS composition, but not overall HS sulfation, suggesting that the HS pattern may be more relevant to CAA development or progression than bulk sulfation levels. We also found certain HS disaccharides (D0H0) associated with APP and other proteins increased in CAA (OLFML3, FRZB, ApoE, FGA), as well as with ApoE genotype, but not with atherosclerosis. Finally, we reveal proteins altered in CAA-affected meninges, including OLFML3 and FRZB, and their association with CAA-specific HS disaccharides. Given that CAA is common and challenging to diagnose antemortem prior to clinically significant vascular disease, OLFML3, FGA, and FRZB may be candidate biomarkers to consider for serum or CSF screens. This multi-faceted understanding of the molecular entities unique to CAA may guide future studies to better understand CAA pathogenesis and to identify antemortem diagnostic markers.

## SUPPLEMENTARY MATERIAL

Supplementary material is available at academic.oup.com/jnen.

## FUNDING

This study was supported by the National Institutes of Health grants NS069566, NS110409, and NS076896 (to C.J.S.), AG061251 (R00) (to P.A.-C.), HL107150 and GM93131 (to J.D.E.), and NIA grant P30 AG062429.

## CONFLICTS OF INTEREST

The authors declare that they have no competing interests.

## Supplementary Material

nlaf018_Supplementary_Data

## Data Availability

The mass spectrometry proteomics data have been deposited to the ProteomeXchange Consortium via the PRIDE^69^ partner repository with the dataset identifier PXD056864.
