# Supplementary material for: Multi-omic analysis of meningeal cerebral amyloid angiopathy reveals enrichment of unsubstituted glucosamine and extracellular proteins
Source: J Neuropathol Exp Neurol. 2025 Mar 29;84(5):398–411. doi: 10.1093/jnen/nlaf018 (PMC12012350; doi:10.1093/jnen/nlaf018)
Supplement: nlaf018_Supplementary_Data [file nlaf018_supplementary_data.zip › nlaf018_Supplementary_Data/Supplementary Figures.docx]

**Supplementary Figures**

**
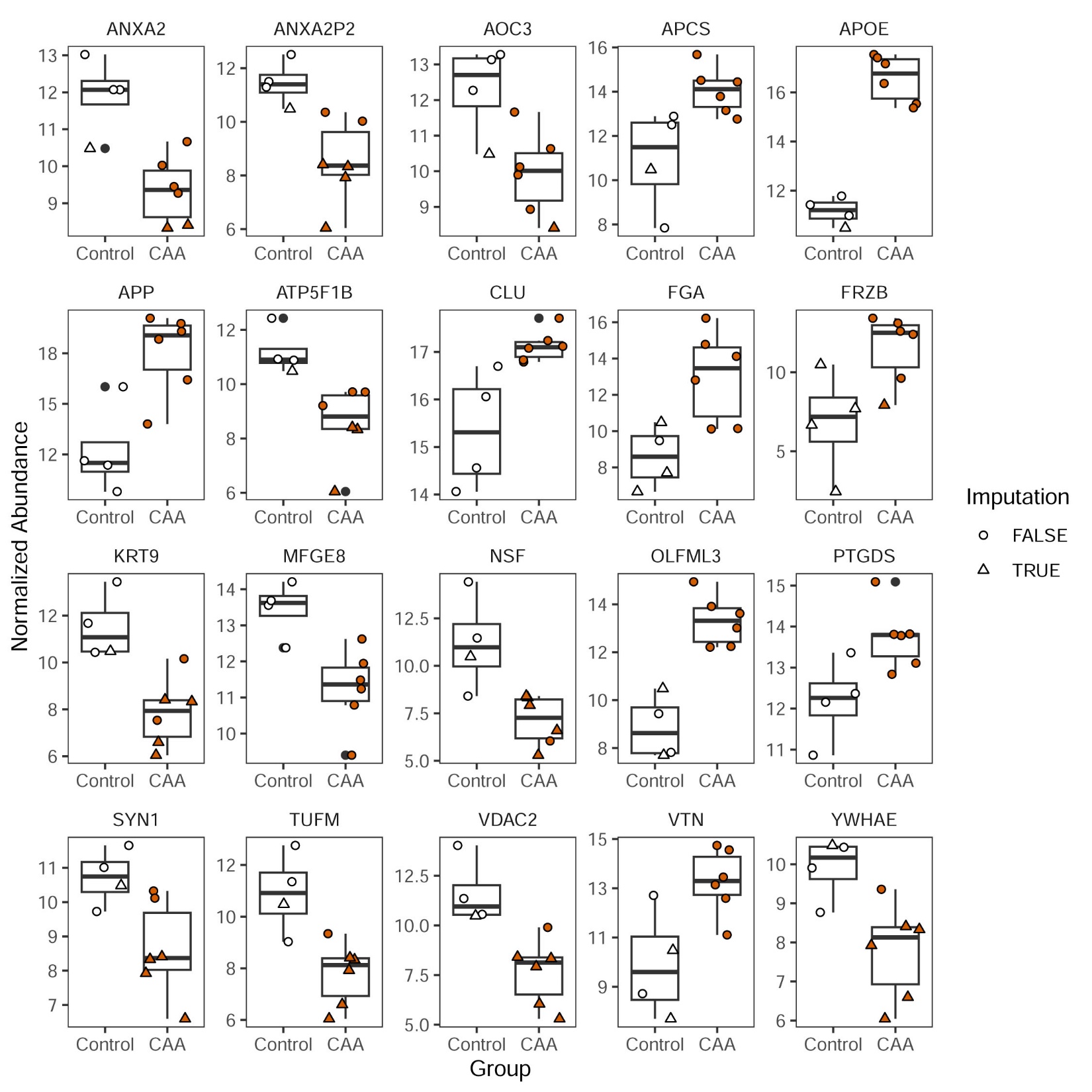
**

**Supplementary Figure 1. Differentially abundant proteins of control and CAA meninges.** Box plots of all differentially abundant genes in control and CAA cases. Values resulting from imputation shown as triangles, non-imputed values shown as circles. Control cases shown as symbols filled with white. CAA cases shown as symbols filled with vermillion. All proteins have a Wilcoxon rank sum test *P*-value less than 0.05. (CAA n = 6, Control n = 4. Values and analysis provided in supplementary table 1, tab 1 “Abundance”).

**
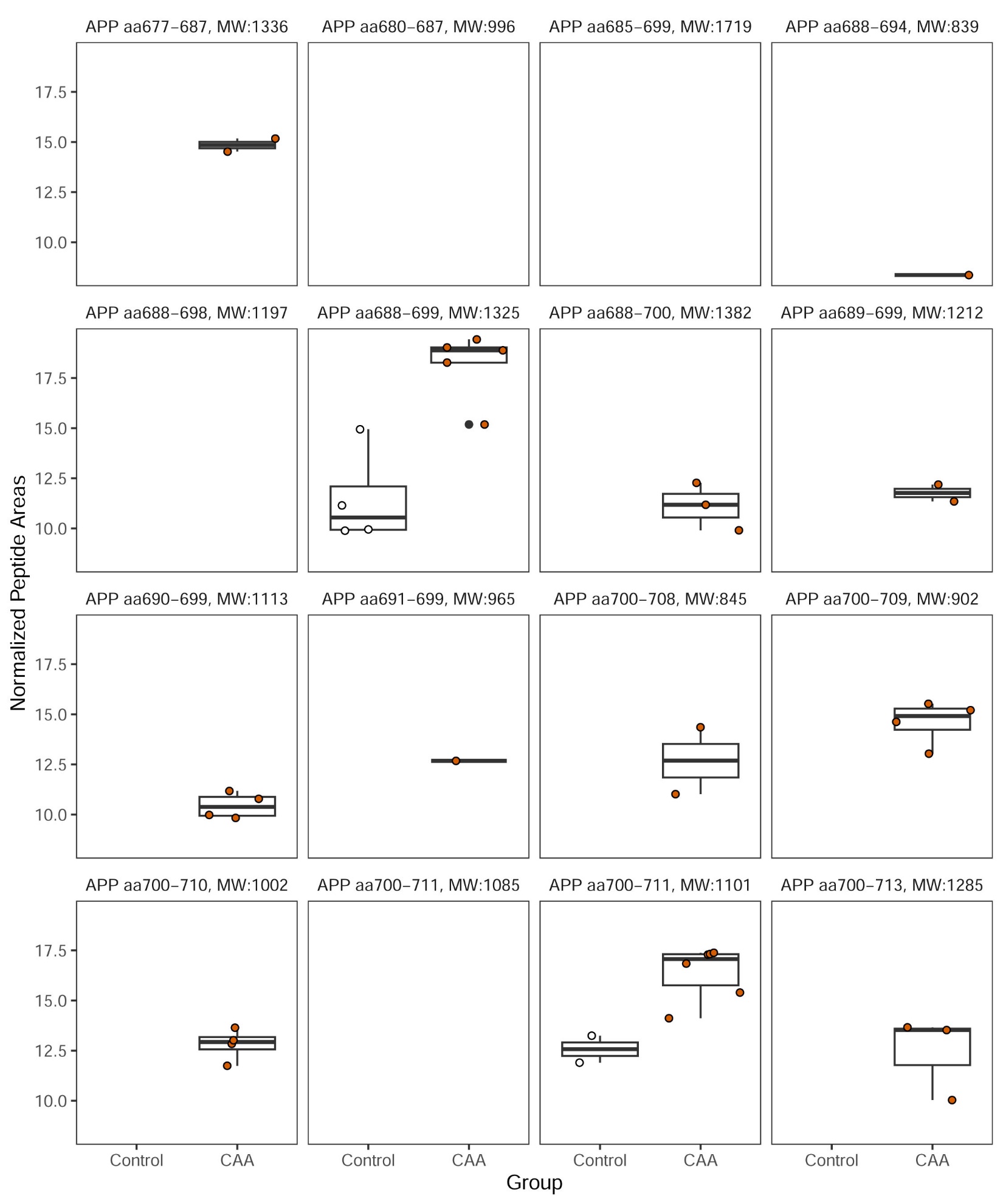
**

**Supplementary Figure 2. APP peptides.** Peptides derived from amyloid beta precursor protein (APP). All detected peptides map to the amyloid beta 40 and 42 (ab40/ab42) region of human APP (amino acids 672-713). Peptide amino acid range and molecular weight (to account for post-translational modification) indicated. Control cases shown as symbols filled with white. CAA cases shown as symbols filled with vermillion. Peptides with no points for either CAA or control were detected in the data set but did not meet criteria for quantitation. A table of all detected APP peptides, their sequences, post-translational modification state, and quantitation are provided on per-sample basis in supplementary table 1, tab 6 “APP Peptides”.

**
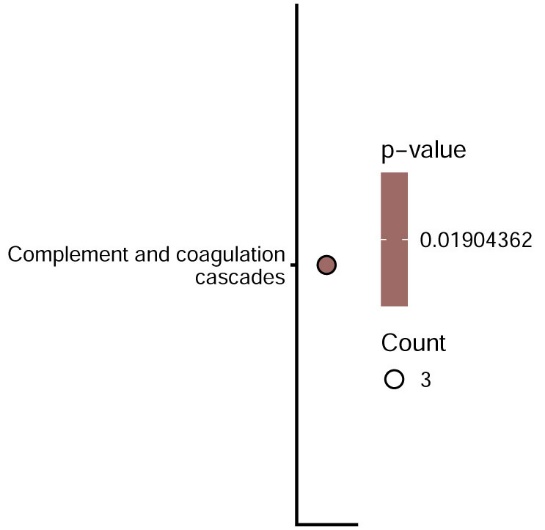
**

**Supplementary Figure 3. KEGG pathway analysis of differentially abundant proteins.**  KEGG pathway analysis of differentially abundant proteins. Adjusted *P*-value indicated by point color. Gene count indicated by point size. All significant ontologies, gene lists, and statistical values provided in supplementary table 1, tab 5 “KEGG Pathway”.

**
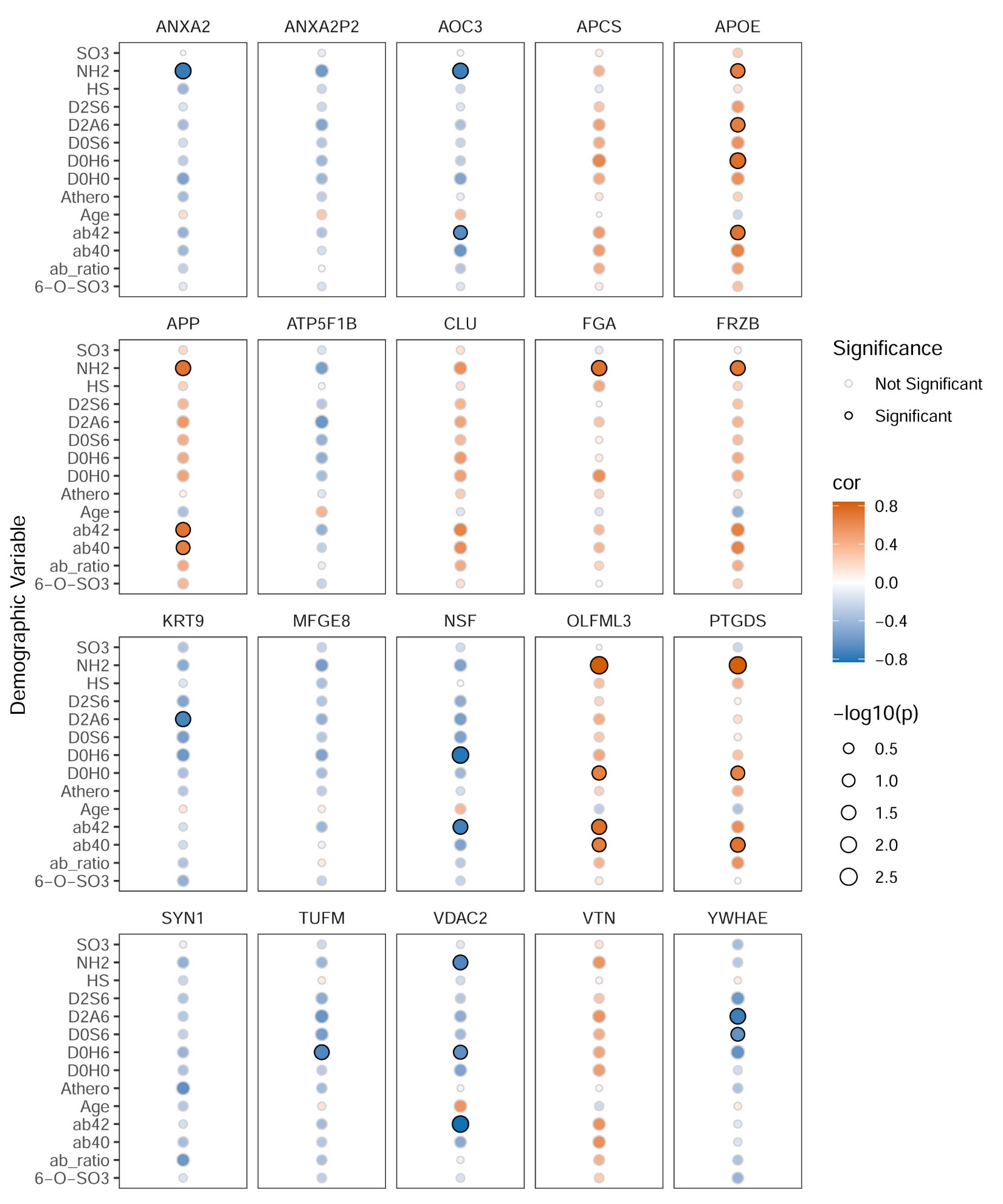
**

**Supplementary Figure 4. Correlation of protein abundance with demographic and HS data.** Visualization of Pearson’s correlation of significantly different protein abundances with demographic data across 10 samples considered in proteomic analysis. Correlation indicated by point color (vermillion = positively correlated, blue = negatively correlated). Point size indicative of Pearson’s correlation *P*-value. Significantly correlated demographics indicated by solid circle around point (Pearson’s correlation *P*-value <= 0.05 was considered significant, n=10). Demographic or HS variables indicated on the y-axis. (SO3 = average sulfate/disaccharide; NH2 = mole percent unsubstituted glucosamine containing disaccharides (includes disaccharides D0H0, D0H6, D2H0, D2H6); HS = micrograms heparan sulfate per gram tissue; D2S6, D2A6, D0S6, D0H6, and D0H0 = mole percent of this disaccharide (abbreviations explained in Supplementary Table 2); Athero = atherosclerosis score; Age = age in years; ab40 = ab40 peptide quantified by ELISA; ab42 = ab42 peptide quantified by ELISA; ab_ratio = ratio of ab40 to ab42 quantified by ELISA; 6-O-SO3 = mole percent hexosamine 6-O-sulfation (includes disaccharides D0H6, D0A6, D2H6, D0S6, D2A6, D2S6).

**
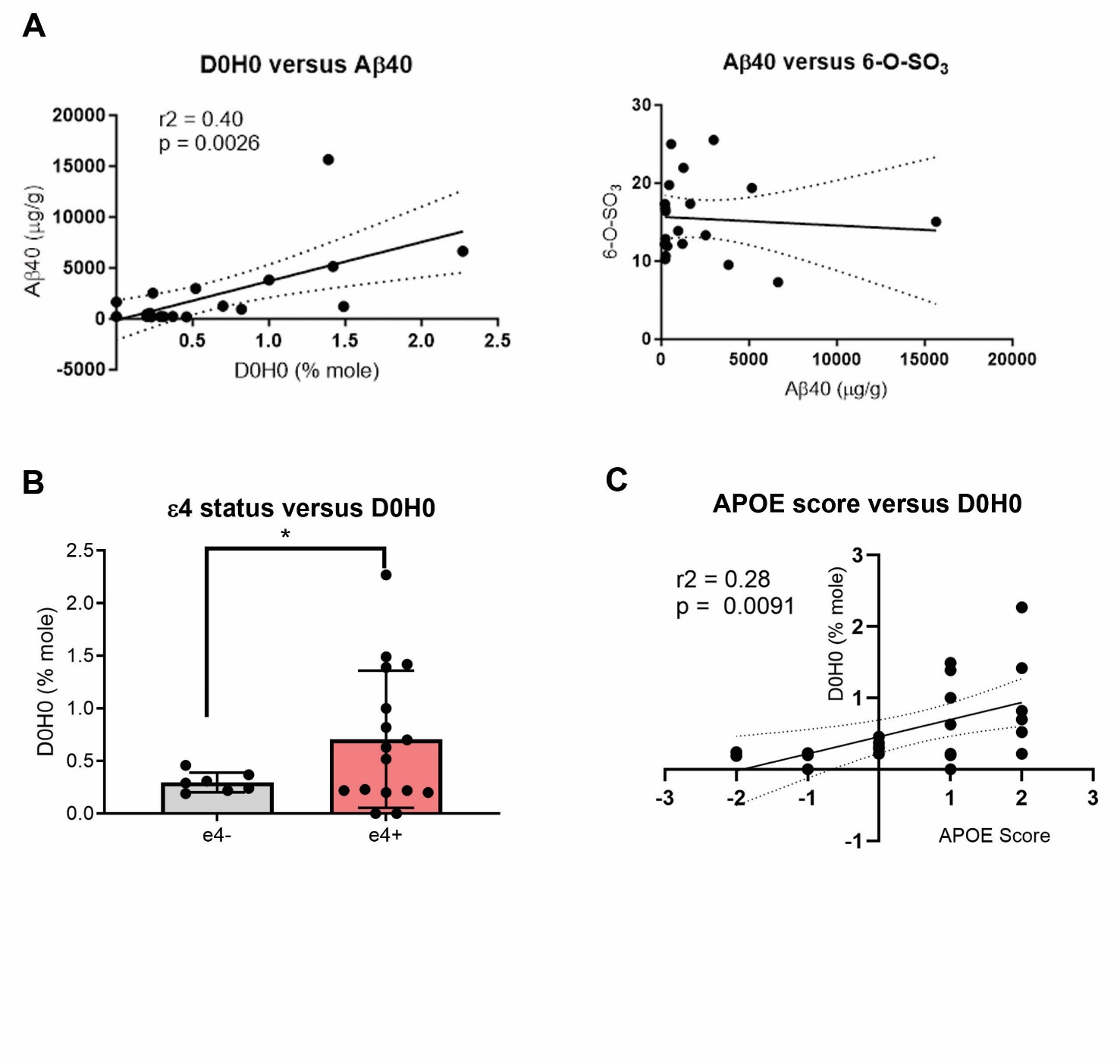
Supplementary Figure 5. Correlation analysis of Aβ40 and APOE genotype with HS disaccharides.** (A)

Simple linear regression analyses indicate that Aβ40 levels correlate with D0H0, but not 6-0-SO_3_ disaccharides. (B) D0H0 levels were increased with possession of ApoE ε4 allele. (C) Simple linear regression analysis of ApoE score (APOE score) reveals a direct relationship between ApoE risk score and abundance of D0H0. Statistics performed in GraphPad Prism software. Significant *P*-values and associated r-squared (r2) values indicated.

**Notes for Supplementary Tables 1 tab 1 “Abundance”.**  Imputed values indicated in vermillion and provided alongside unmodified quantitation and normalized quantitation.
