# Supplementary material for: Multi-omic analysis of meningeal cerebral amyloid angiopathy reveals enrichment of unsubstituted glucosamine and extracellular proteins
Source: J Neuropathol Exp Neurol. 2025 Mar 29;84(5):398–411. doi: 10.1093/jnen/nlaf018 (PMC12012350; doi:10.1093/jnen/nlaf018)
Supplement: nlaf018_Supplementary_Data [file nlaf018_supplementary_data.zip › nlaf018_Supplementary_Data/Supplementary Table 2.docx]

| **Abbreviation** | **Structure** |
| --- | --- |
| D0H0 | ΔUA-GlcNH2 |
| D0A0 | ΔUA-GlcNAc |
| D0H6 | ΔUA2S-GlcNH26S |
| D2H0 | ΔUA2S-GlcNH2 |
| D0S0 | ΔUA-GlcNS |
| D0A6 | ΔUA-GlcNAc6S |
| D2A0 | ΔUA2S-GlcNAc |
| D2H6 | ΔUA2S-GlcNH26S |
| D0S6 | ΔUA-GlcNS6S |
| D2S0 | ΔUA2S-GlcNS |
| D2A6 | ΔUA2S-GlcNAc6S |
| D2S6 | ΔUA2S-GlcNS6S |

**Supplementary Table 2. Disaccharide abbreviation table.** Disaccharide abbreviation and corresponding structure base on nomenclature proposed by Lawrence et al., 2008 (https://doi.org/10.1038/nmeth0408-291).
