# Supplementary material for: Multi-omic analysis of meningeal cerebral amyloid angiopathy reveals enrichment of unsubstituted glucosamine and extracellular proteins
Source: J Neuropathol Exp Neurol. 2025 Mar 29;84(5):398–411. doi: 10.1093/jnen/nlaf018 (PMC12012350; doi:10.1093/jnen/nlaf018)
Supplement: nlaf018_Supplementary_Data [file nlaf018_supplementary_data.zip › nlaf018_Supplementary_Data/Supplementary Table 3.docx]

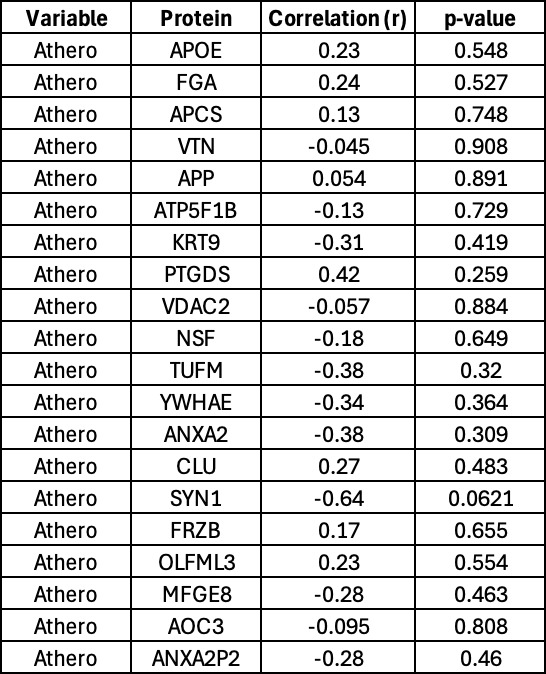


**Supplementary Table 3. Correlation of significantly different proteins with atherosclerosis.** No statistically altered proteins demonstrate a statistically significant correlation with atherosclerosis characterized at autopsy.
