# Supplementary material for: Multi-omic analysis of meningeal cerebral amyloid angiopathy reveals enrichment of unsubstituted glucosamine and extracellular proteins
Source: J Neuropathol Exp Neurol. 2025 Mar 29;84(5):398–411. doi: 10.1093/jnen/nlaf018 (PMC12012350; doi:10.1093/jnen/nlaf018)
Supplement: nlaf018_Supplementary_Data [file nlaf018_supplementary_data.zip › nlaf018_Supplementary_Data/Supplementary Table 4.docx]

| Dependent variable^ | r^2^ | Regression  *P*-value | Ɛ4 dose (0,1,2)  *P*-value | Aβ40*  *P*-value |
| --- | --- | --- | --- | --- |
| NH_2_ | 0.67 | 0.0015 | 0.035 | 0.0027 |
| D0H0 | 0.52 | 0.0072 | 0.18 | 0.0051 |
| 6-SO3 | 0.21 | 0.13 | 0.050 | 0.38 |
| D0S6 | 0.44 | 0.023 | 0.010 | 0.36 |
| D2A6 | 0.58 | 0.0027 | 0.001 | 0.53 |
| D2S6 | 0.21 | 0.27 | 0.16 | 0.98 |

^n = 12 CAA and n = 8 control cases

*Total Aβ40 (FA + SDS)

**Supplementary Table 4.** The relationship between Aβ40 level and ApoE genotype with HS composition by multivariate regression analysis.
